# The Indian National Association for Supplying Female Medical Aid to the Women of India, and the Countess Dufferin's Fund

**Published:** 1888-12-08

**Authors:** William Moore

**Affiliations:** K.C.I.E., late Surgeon-General with the Government of Bombay


### The Indian National Association for supplying Female Medical Aid to the Women of India, and the Countess Dufferin's Fund.

By Sir William Moore, K.C.I.E., late Surgeon-General with the Government of Bombay.

As the result of heredity and education, and partly as the result of climate, Eastern nations regard many subjects in a different light from that in which we, who pride ourselves on Western civilisation, view such subjects. There is, however, no characteristic of Eastern nations more prevalent than the lower value they set upon females. Neither is this of modern origin, nor entirely confined to the remote East. Plato said the female is doubtless the inferior. Pericles said of the Athenian women, they were best who were least heard of. Even old Herodotus sneers at females as "mere chattels." But the worst emanates from an ancient Eastern sage, who gave his opinion thus: "From 10 to 20 years of age, fair to the eye of the beholder, a phrase corresponding with 'beaute du diable'; from 20 to 30, still fair and full of flesh; from 30 to 40, a mother of many boys and girls; from 40 to 50, an old woman of the deceitful; from 50 to 60, slay her with a knife; from 60 to 70, the curse of Allah upon them one and all." But the people of India were never quite so much disposed to degrade females into beings of an inferior order as some other nations. Thanks to the extension of European civilisation, and the adoption by Indians of various European customs, Indian women are daily taking a higher place, and receiving an education to which even their mothers were strangers. Yet, notwithstanding this, there always was, and now is, an intense dislike, amounting in many families to absolute prohibition, for the females to be even seen by any male not belonging in a very near degree to the family. Hence it used to be that a doctor, whether European or Indian, rarely looked on an Indian female of the class referred to, however serious her ailment might be. Occasionally European medical men were called in, but in the great majority of cases too late for their services to prove beneficial. Often they were expected to diagnose the disease, and prescribe the remedy, without even seeing more of the patient than her hand thrust through a slit in a curtain, so that the pulse might be felt. It must not, however, be supposed that no Indian females were ever seen by male doctors, for the female out-patient departments of Indian hospitals are thronged, and the number of females in most of the large hospitals is only limited by the number of beds available. But such female patients are chiefly low-caste women, who if they have the desire certainly have not the means to become what is called *perdahnisheen*, or "behind the curtain." When a woman is "perdah" she cannot be seen by any male excepting those of her own family. "Perdah" women, both Mahomedan and Hindu, constitute a very large proportion of the female population of India, more so in some districts than in others. And if the women themselves, as there is reason to believe is often the case, were willing in sheer extremity to see a male doctor, the men make it a point of honour to object. Hence suffering and disease proceeds unrelieved and unchecked in very many of the households of India. It may be truly stated that hundreds of thousands of Indian females "have died of medicable wounds." Not only during ordinary ailments to which all womenkind are liable; but also "when epidemics rush as a storm o'er the astonish'd land, and strew with sudden carcases the ground," the only treatment available for the Indian "perdah" female is that accorded by some illiterate female wise woman or quack. The treatment lying-in women receive is cruel as extraordinary. The "Dhais," or women who attend at such occasions, maltreat their victims through ignorance, through carelessness, and, as there is ample reason to believe, through malicious intent. The worst room in the house, a charcoal fire, an utter absence of ventilation, a crowd of female spectators, accumulations of all kinds of filth, are among the least evils to which lying-in women are subjected, the result being the prevalence of puerperal fever and tetanus, and if these evils are escaped, various diseases of the womb afterwards.

Some years back a society was instituted in Bombay chiefly through the exertions of Mr. Ketteridge, for the supply of female medical aid for Indian women. But shortly afterwards the Countess of Dufferin arriving in India, took up the matter, and instituted, on unsectarian basis, "The National Association for Supplying Female Medical Aid to the Women of India." Her Majesty the Queen-Empress graciously consented to be patron, while the governors and lieutenant-governors of the various Indian provinces became vice-patrons. In the formation of the Association and in its progress to the present time, great assistance was received fron Lady Reay in Bombay, from Lady Lyall in the North-West Provinces, and from Lady Aitcheson. The Association consists of life councillors, of life members, and of ordinary members, according to amount of donation, and among the different classes are many Indian gentlemen. The Association is worked by a central committee and branch committees. The money subscribed is known as the "Countess of Dufferin's Fund," and to the Central Fund, up to a few weeks back, was subscribed some seven lakhs of rupees, or taking the rupee at the old value, about £70,000. Of this 5½ lakhs have been invested, and out of the income thus obtainable, six medical, twelve nursing, and two hospital assistant scholarships have been permanently established. Grants in aid to various local funds, to hospitals having female wards, and for the translation of medical works, have also been made. It will therefore appear that the fund has been managed very economically, the intention being not to spend means in the erection of new palatial hospitals, but to utilize as much as possible existing establishments by additions of accommodation and staff suitable for female patients. The only connexion the Government of India have had with the Association, or with the fund, is the accord of permission for Government medical officers to report on the progress of Association medical institutions, which assistance has been freely and gladly rendered; and an intimation to municipalities that a portion of the funds at their disposal for medical purposes, might legitimately be devoted to the aid of female hospitals and dispensaries.

One of the principal objects of the Association is to supply females educated in medicine, and it has been determined to provide three classes: First, lady doctors possessing such qualifications as would entitle them to register under the English Act; secondly, female assistant surgeons; and thirdly, female hospital assistants; the designations of the latter being the same, with the addition of the prefix "female," as the grades of male medical officers in the employ of Government, while the curriculum of instruction is to be altogether the same. At present the lady doctors must be procured from England or America, the qualification, in addition to the diploma, being a certificate of health and fitness for service. The honorarium may be estimated at 350 rupees per mensem, with liberty of private practice in the locality to which they may be accredited. It is, however. only by educating females of the country that the wants can be effectually and eventually met—women born in the country versed in its customs, and belonging to the people. The commencement has been made. Nearly two hundred girls are now being educated in the medical schools of India, the majority for the lower grade of female hospital assistants, which, however, entails a three years' course of instruction. But at the Grant Medical College of Bombay, twenty-two young ladies have been, or are being educated for the higher grades of lady doctor, or assistant surgeon. Madras was the first place in India where female students were received into the medical school, and amongst successful Madras students was Mrs. Scharlieb, who now fills a medical office in London. At the present time there are fifteen lady doctors employed in India in connxtion with the Countess of Dufferin's Fund. There are female hospitals at Agra, Calcutta, Durbungha, Hyderabad - Deccan, Hyderabad - Sind, Lahore, Madras, Mysore, Nagpore, Odeypore, Ranpoor, and Ulwur, and fifteen female dispensaries at the same or at other places. Some as at Odeypore and Ulwur, are supported by Indian princes. The list does not include the Cama Hospital for Women, in Bombay, built by the liberality of the Cama family, supported by Government and ably superintended by Miss Dr. Peechey, aided by two other lady doctors; nor the Victoria Female Hospital, at Madras, also supported by Government.

The provision of hospitals and dispensaries, and of female medical officers for such institutions, is not the only aim of the "National Association for Supplying Female Medical Aid to the Women of India," or the only purposes to which the funds are applied. The education of midwives and nurses has not been neglected. It has been already stated that twelve nursing scholarships have been instituted, and classes for the teaching of both nurses and midwives have been formed at Agra, Lahore, Calcutta, Bombay, Poonah, and other places. That competent midwives are much required is evident from what has been previously remarked on the treatment of lying-in women; and although it may be long before a competent body of midwives replaces the ignorant "Dhais" who now so ill perform their part, still there is hope that, at least in the large centres, the "Dhais" may soon find their occupation gone, as is the case with the "baids," "hukeems," and "jarrahs," the Indian physicians, apothecaries, and surgeons of former days, who have been superseded by the natives of India, educated in the medical colleges under European supervision. It is true that somewhat desultory attempts have been made in this direction previously. A class for the education of native females as midwives was in operation some years back at the Medical College in Bombay, but owing to various circumstances it did not succeed. The fact is, the Dhais were preferred; for it takes long to break down old customs, especially when supported by ignorance, superstitution, and vested interests. As for nurses, those educated at all found ample employment in existing hospitals. The present time is more favourable for the outside employment of midwives and nurses than any previous period; for, owing to the exertions of the Countess Dufferin and her lady coadjutors, the attention of the whole community has been aroused to the subject—a community becoming yearly more fitted by the extension of education among all classes, to appreciate any movement conducing to their own benefit.

The above only relates to the "Countess Dufferin's Central Fund." But the branch funds have been equally more or less successful. For instance, the Bombay branch fund, supervised by Lady Reay, has made several grants for important purposes; amongst them for building quarters for probabioner nurses and midwives at the Cama female Hospital in Bombay; also 5,000 Rupees towards the construction of quarters for the lady doctors in charge of the obstetric department of the Cama Hospital.

Very recently, Lady Dufferin has written an account of the special work of the Central Committee, over which she has presided for the last three years. Lady Dufferin closes her memorandum with an eloquent appeal to the men of India as follows:

"The National Association, and all those who have subscribed to it, those who have worked for it, those who are now striving to teach, and to train, and to relieve the sick, have done and are doing what they can for the women of this country, but if their work is to be a universal one, if relief is to be brought not to tens, but to hundreds of thousands of Indian homes, as it should be, then it is not one society, or a certain number of single individuals who can accomplish such a task. It is the determined attitude of the men of this country which must do it. It lies with them to give the women dependent upon them relief in suffering, to save them from a kind of professional aid, which is oftentimes destruction, and to introduce into their households those sanitary regulations, and that intelligent management of women and children which saves life and promotes good health."

As before observed, there is no doubt that the women of India are held in higher estimation than was formerly the case, and it may be hoped that the examples of the Indian Princes (especially Jeypoor, Odeypoor, and Ulwar) who have subscribed largely to the fund, or who have established and support female hospitals, will be followed by the masses. For it is from the bulk of the people that the fund must be eventually supported. That the "Purdah" women of India are thankful for the strenuous attempt which has been made for their benefit is evident from an address recently submitted by the "Purdah-nisheen" ladies of Lahore to the Countess Dufferin: "We take great pleasure in thinking that the prejudices against medical treatment on the European lines which were at one time rampant amongst us are rapidly disappearing before the progress of scientific education in this country, and that at no distant date adequate female medical skill will be abundantly available to the Indian women, whose beds of sickness, even in very serious feminine ailments, no male doctor can approach. . . . We are very grateful to your Excellency for having afforded us this opportunity of expressing our deep, sincere, and heartfelt gratitude . . ." And there is no doubt that before leaving India, the Countess Dufferin will receive many such addresses.

